# Lives of British Physicians
*Family Library.


**Published:** 1830-10-01

**Authors:** 


### XXIV.

### LIVES OF BRITISH PHYSICIANS.*

WE intend to extract an anecdote or two occasionally from this amusing little volume. It must always be interesting to the present generation of medical men, to gain something like an insight into the private life and the chimney-corner of those who have long since earned their share of wealth and reputation, and passed away from the busy stage. Surely nothing impresses on the mind, with more solemn force, the futility and vanity of man's fretful and chequered career, than the study of biography! It is the same with all, the PRINCE and the clown—the man of talent, and the blockhead! We read that, on such a day, in such a year, they were born;—that they engaged in certain pursuits, and then—they died. But our mood must not be sad:—we took up the pen for amusement, perchance instruction; and why should we dip it in the ink of despondency!

### DR. WILLIAM HUNTER.

The first eminent man to whom we direct our attention, is Dr. WILLIAM HUNTER. How strange and how awful the contrast between his first lecture and his last.

"His first anatomical course was attempted in 1746. He experienced much anxiety and doubt at the outset, but applause gradually inspired him with confidence, and he at length found the principal happiness of life to consist in the delivery of a lecture. Mr. Watson, one of his earliest pupils, accompanied him home after the trying moment of his introductory discourse. Hunter had just received seventy guineas from admission-fees, which he carried in a bag under his cloak, and observed to his friend, that it was a larger sum than he had ever before possessed. The early difficulties of eminent men form perhaps the most instructive and animating portion of their biography. Linnæus records of himself, *Exivi patria triginti sex nummis aureis dives*. The profits of his two first courses were considerable; but, by contributing to relieve the wants of some of his friends, he found himself, on the approach of the third season, under the necessity of deferring his lectures for a fortnight, merely from the want of money to supply the expense of the usual advertisements. This unpleasant embarrassment operated as a check upon him in the use of money, and probably formed one remote source of the large fortune which he afterwards accumulated."

All here was buoyant; success converted the pale tremors of diffidence and suspense into the full flush and swing of conscious vigour. As happens with professional men, he lived but in the atmosphere of his own creation; he was miserable, save when lecturing. At last, like the old and gallant steed, on whose ears have burst the well-known notes of the trump and the bugle, he rushes to the charge, exults, and dies.

"About ten years before his end his health was so much impaired, that, fearing he might soon become unfit for the profession which he loved, he proposed to recruit himself by a residence in Scotland, and was on the eve of purchasing a considerable estate, when

* Family Library.

the project was frustrated by a defect in the title-deeds. This trifle banished his rural plans, and he remained in London, continually declining in health, but pursuing distinction with the same ardour with which he had courted it in his earlier days. He rose from a bed of sickness to deliver an introductory lecture on the operations of surgery, in opposition to the earnest remonstrances of his friends. The lecture was accordingly delivered, but it was his last; towards the conclusion his strength was so much exhausted, that he fainted away, and was finally replaced in the chamber which he had been so eager to quit. In a few days he was no more. Turning to his friend Combe, in his latter moments, he observed, '*If I had strength enough to hold a pen, I would write how easy and pleasant a thing it is to die.*' He expired on the 30th of March, 1783: his brother, John, occasionally introduced the cathether in this last paralytic seizure."

Dr. Hunter, it is well known, confined himself to the practice of midwifery, and the following statement respecting the earlier teachers of this branch of medicine, may probably be gratifying to Sir Anthony Carlisle.

"Mawbray seems to have been the first teacher of obstetrics in London. He was lecturing in 1725, and established a lying-in hospital, to which students were admitted. The Chamberlains followed him—a family which professed to possess a better method of treating difficult labours than was known to others, and maintained a sort of mystery as to their instruments. This pretension was imitated by others. Smellie gave a new dignity to the subject by his talents and his lessons; although he is accused by a rival of advertising to teach the whole science in four lectures, and of hanging out a paper lantern, inscribed with the economical invitation, "*Midwifery is taught here for five shillings!*"

When Dr. Hunter invited his younger friends to his table, they were seldom regaled with more than two dishes; when alone he rarely sat down to more than one: he would say, "*A man who cannot dine on this deserves to have no dinner.*" After the meal, his servant (who was also the attendant on the anatomical theatre) used to hand round a single glass of wine to each of his guests. How different all this from the modes of the day! Now, if a few pupils dine with their teacher, a dinner in state, with claret and champagne, is scarcely considered good enough for the nice young gentlemen. Our modern instructors have discovered, that, provided they appeal in a touching style to the belly, they need give themselves comparatively little trouble respecting the brains of students.

### DR. BAILLIE.

So much has been written on this honoured name, and his memory is yet so green in the memory of all, that we shall only select one short anecdote of his irritability when much pressed by business. We have seen it before, but perhaps it may be new to many of our readers, and it possesses the inestimable advantage of brevity.

"During his latter years, when he had retired from all but consultation practice, and had ample time to attend to each individual case, he was very deliberate, tolerant, and willing to listen to whatever was said to him by the patient; but, at an earlier period, in the hurry of great business, when his day's work, as he was used to say, amounted to sixteen hours, he was sometimes rather irritable, and betrayed a want of temper in hearing the tiresome details of an unimportant story. After listening, with torture, to a prosing account from a lady, who ailed so little that she was going to the Opera that evening, he had happily escaped from the room, when he was urgently requested to step up stairs again; it was to ask him whether, on her return from the Opera, she might eat some oysters: 'Yes, Ma'am,' said Baillie, 'shells and all.'"

### DR. JENNER.

Jenner was certainly a fortunate man. He lived to see his doctrines enthusiastically embraced; he was styled the benefactor of mankind, and what was still better, he knew that he was so; he received a handsome parliamentary grant; and he died before those manifold failures of vaccination had started into any thing like a prominent attitude. It is true that his rewards were far, very far disproportioned to his merits; but still, when we look at the fate that commonly awaits discoverers, we cannot but consider JENNER as pre-eminently fortunate. The biographer of this amiable and great physician takes a less encouraging view of this subject.

"Nevertheless," says he, "a painful reflection is forced upon us, in considering the history of Jenner; he surely did not receive, among his countrymen, the distinction, the fortune, and the fame which he merited. It seems that, among nations called civilized, the persons who contribute to amusement, and to the immediate gratification of the senses, occupy a higher share of attention, than the gifted and generous beings who devote their existence to the discovery of truths of vital importance. The sculptor, the painter, the musician, the actor, shall engross, a thousand times, the thoughts of citizens who, perhaps, only five times in a whole life, consider the merits of a Jenner. The little arts of puffing, the mean machinery of ostentation, never once entered the heads of a Newton, a Watt, or a Jenner; but they protrude into meridian splendour the puny pretensions of countless poetasters, witlings, and amateurs. Real genius and active industry should not be dismayed, however, by this indifference which clouds the dawn of their exertions, and which sometimes nips the bud of noble aspirations; for great truths there will always come a time and a place; the man who works for the benefit of his fellow-beings can afford to await the hour allotted for the full development of his labours, and bequeaths, in tranquil confidence, to posterity the reputation which he may have failed to obtain from a dominant coterie of capricious contemporaries."

Those who can look philosophically through the mist of years, and, like Swift, dedicate their lives to PRINCE POSTERITY, may scorn the little buzz of ephemeral popularity. For those, however, who have no such noble yearnings after posthumous fame, it is mortifying, no doubt, to see the world more disposed,—

"To worship Catalani's pantaloons, than pay the eager tribute of admiration to solid unpretending worth.

We may mention of Jenner, ere we part, that, before he had matured his magnificent discovery, for such it deserves to be called, the Alveston Medical Club were so bored with him and it, that they threatened to expel him. When about to publish his first memoir, he was seriously admonished not to present it to the Royal Society, lest it should *injure* a character acquired by some observations on the CUCKOO! If these examples are not sufficient to shew the blindness of man, and the hair-breadth 'scapes to which the most beneficial improvements are liable, then will we—

"Break our pipe and never whistle mair."

DR. GOOCH.

Alas, poor Gooch! Too frail and too irritable for this world, thou art quietly reposing at last with the wife of thy bosom and the children of thy affections, in the silence of the tomb! Thy faults, and they were few, are wrapped in the shroud;—thy excellencies survive for thy family and thy profession. We recommend the life of Gooch to the perusal of all, who can look, with saddened and subdued gratification, on genius and talent and enterprise, struggling through a feeble corporeal frame, a melancholic temperament, and unremitting ill health.

P. S. The Editor, who is not the writer of the foregoing notice of Dr. Gooch's biography, may be permitted to state that he was on terms of intimacy with Dr. Gooch for five or six years before his death. The memoir, though very minute, perhaps prolix, on many points, is not complete. Dr. Gooch consulted the Editor of this Journal more than once respecting a complaint which harrassed him greatly, though not at all alluded to in the memoir—viz. a prolapsus ani and a considerable discharge from the rectum. Dr. Gooch was a HYPOCHONDRIAC, in consequence of the state of his stomach

—and somewhat of an enthusiast, at the same time, which accounts for many of the sentiments of despondency and sanguine expectations blended in his correspondence with Mr. Southey. It has been already mentioned in a former number of this Journal, that the amiable and expectant deceased consulted the Editor, when within a few days of his death, respecting a journey to Nice or Pisa, for the prolongation of his life, if not the restoration of his health!! This shewed the predominancy of the pulmonary over the gastric disease, towards the close of life—a predominancy which fortunately strewed the path to the tomb with flowers, and verified the adage that—

"Hope springs eternal in the *hectic* breast."

